# Primate immune responses to HIV-1 Env formulated in the saponin-based adjuvant AbISCO-100 in the presence or absence of TLR9 co-stimulation

**DOI:** 10.1038/srep08925

**Published:** 2015-03-12

**Authors:** Paola Martinez, Christopher Sundling, Sijy O'Dell, John R. Mascola, Richard T. Wyatt, Gunilla B. Karlsson Hedestam

**Affiliations:** 1 Department of Microbiology, Tumor and Cell Biology, Karolinska Institutet, Stockholm, Sweden; 2 Vaccine Research Center, National Institute of Allergy and Infectious Diseases, National Institutes of Health, Bethesda, MD, USA; 3 Department of Immunology and Microbial Science, IAVI Neutralizing Antibody Center, The Scripps Research Institute, La Jolla, CA USA

## Abstract

Protein-based vaccines require adjuvants to achieve optimal responses. Toll-like receptor (TLR) 9 agonists were previously shown to improve responses to protein-based vaccines, such as the Hepatitis B virus vaccine formulated in alum. Here, we used CpG-C together with the clinically relevant saponin-based adjuvant AbISCO-100/Matrix-M (AbISCO), to assess if TLR9 co-stimulation would quantitatively or qualitatively modulate HIV-1 envelope glycoprotein (Env)-specific B and T cell responses in rhesus macaques. The macaques were inoculated with soluble Env trimers in AbISCO, with or without the addition of CpG-C, using an interval similar to the Hepatitis B virus vaccine. Following a comprehensive evaluation of antigen-specific responses in multiple immune compartments, we show that the Env-specific circulating IgG, memory B cells and plasma cells displayed similar kinetics and magnitude in the presence or absence of CpG-C and that there was no apparent difference between the two groups in the elicited HIV-1 neutralizing antibody titers or antigen-specific CD4+ T cell responses. Importantly, the control of SHIV viremia was significantly improved in animals from both Env-immunized groups relative to adjuvant alone controls, demonstrating the potential of AbISCO to act as a stand-alone adjuvant for Env-based vaccines.

An improved understanding of vaccine-induced B cell responses in primates is required to accelerate the development of new and effective prophylactic vaccines for humans, including one against HIV-1. A majority of modern day anti-viral vaccines are based on highly purified recombinant protein antigens, which require co-administration with an adjuvant to evoke a high-titer immune response^1^,^2^,^3^. The extent to which different vaccine adjuvants promote the establishment of peak as well as long-lived immune responses to protein antigens is at present insufficiently understood. To prioritize adjuvant formulations, side-by-side comparisons and longitudinal examination of elicited responses are required.

Prior reports suggest that the addition of Toll-like receptor (TLR) agonists to some vaccines formulated in TLR-independent adjuvants, such as alum, qualitatively and/or quantitatively improves the induced immune responses. For example, addition of CpG oligonucleotides (ODN) to stimulate TLR9 signaling increased hepatitis B virus-specific Ab titers^4^ and enhanced Ab affinity maturation^5^ in Engerix-B vaccinated humans. More modest effects were observed when CpG ODN was administered together with the otherwise non-adjuvanted split detergent Flu vaccine, Fluarix^6^, or with the *Plasmodium falciparum*-based alum-formulated vaccine, AMA1^7^. However, several parameters differed between these studies, including the immunization interval and the nature and dose of the antigen. Thus, a general conclusion about the effect of TLR9 stimulation on antigen-specific B cell responses could not be drawn from these studies.

TLR9 ligands can be classified into three classes, CpG-A, CpG-B and CpG-C based on their distinct chemical and biophysical properties. CpG-A consists of palindromic unmethylated DNA sequences that form aggregates, while CpG-B does not contain palindromic sequences and remains soluble^8^. The two classes of CpG also have different chemical backbones: CpG-A has a phosphodiester sequence with phosphorothioate G-rich ends and CpG-B has a phosphorothioate backbone. CpG-A was previously shown to be retained a longer time in early endosomes leading to more efficient activation of the MyD88-TRAF6-IRAK1-IRF7 pathway and subsequent IFN-α production in PDCs^9^, while CpG-B is a potent B cell stimulator^8^. CpG-C shares characteristics of both CpG-A and CpG-B.

In addition to TLR9 ligands, several studies describe the evaluation of other TLR ligands used either alone or in combination with other adjuvant formulations^10^,^11^,^12^,^13^,^14^. General effects of TLR stimulation include rapid infiltration of innate cell types to the site of injection, cytokine production and activation of antigen-presenting cells^14^, ultimately leading to the induction of antigen-specific Ab and T cell responses. However, the specific effects of TLR stimulation varies for different antigens, different formulations, and for the particular TLR ligand used. In-depth analysis, under well-controlled conditions, of specific antigen-adjuvant combinations is therefore required to determine the effects of TLR co-stimulation.

To achieve this, studies in pre-clinical animal models are needed. Macaques provide a highly relevant model system for these questions as many aspects of the immune system are similar between macaques and humans, such as the overall genetics^15^, the characteristics of dendritic cell (DC), B and T cell subsets^16^,^17^ and the family distribution of individual antibody V(D)J gene segments^18^. There are also similarities between humans and macaques with regard to the heterogeneity of MHC alleles and responsiveness to immunization^19^,^20^. Further, for HIV-1-based vaccine candidates, macaques represent the most appropriate model for evaluating protective immune responses, since they can be infected with chimeric simian immunodeficiency viruses (SIV)-HIV-1 (SHIVs) following vaccination with a given candidate vaccine.

In our previous Env immunogenicity experiments in non-human primates^17^,^21^, we used AbISCO in combination with CpG-C ODN to promote humoral as well as cellular immune responses. This adjuvant combination was chosen based on reports showing that synergistic effects were obtained by using the related saponin-based adjuvant ISCOMATRIX in combination with CpG ODN, in both mice and primates^4^,^22^,^23^. Here, we wished to directly determine the effect of TLR9 co-stimulation in the context of AbISCO-formulated vaccines and we therefore immunized rhesus macaques with purified trimeric gp140-F Env glycoprotein in AbISCO in the presence or absence of the TLR9 ligand CpG-C. We show that the induced responses were similar in both groups suggesting that CpG-C did not measurably enhance or modulate the responses stimulated by Env in AbISCO alone. In support of this, we observed a similarly improved control of viremia in SHIV-infected animals in both groups of vaccinated animals compared to the non-vaccinated animals, demonstrating the potential to design effective immunization regimens based on recombinant Env glycoproteins administered in AbISCO.

## Results

### The kinetics and magnitude of Env-specific IgG were not enhanced by TLR9 co-stimulation

**Figure 1:**
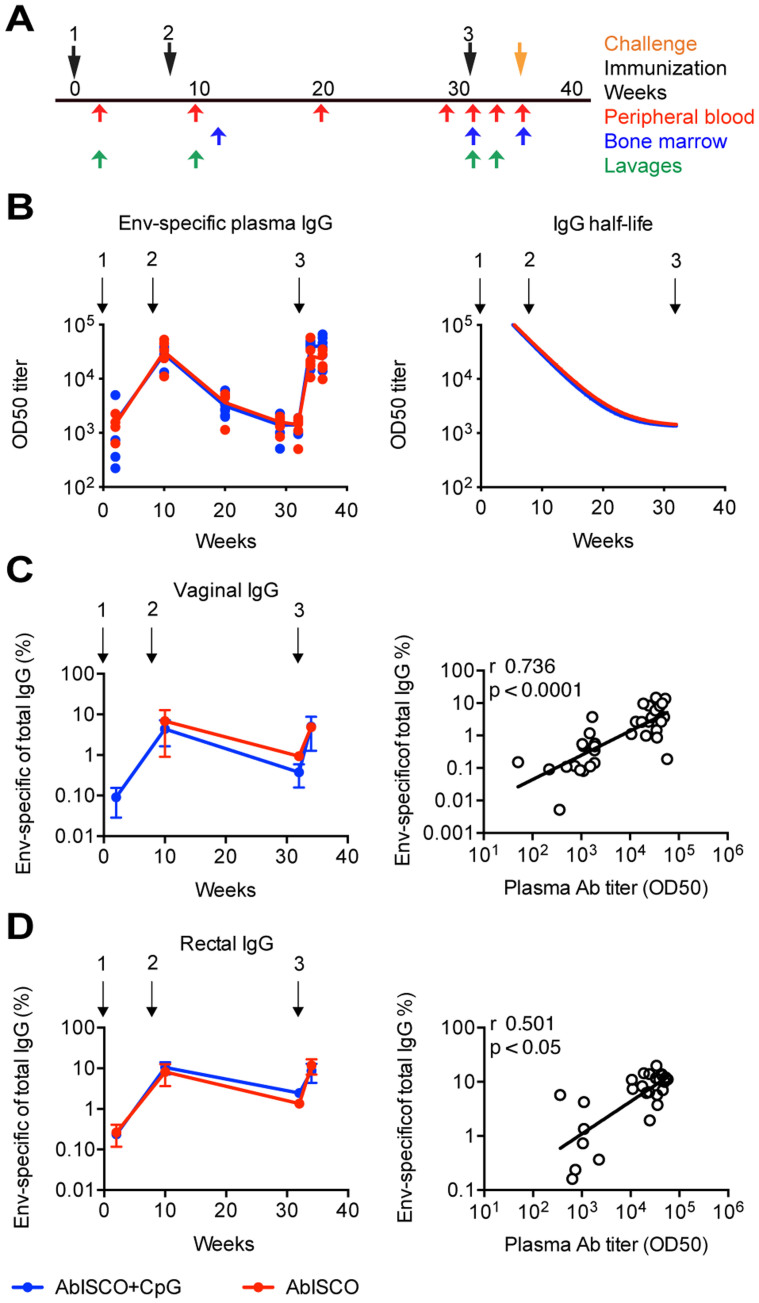
Kinetics of the Env-specific IgG response in periphery and mucosa after immunization. Animals were divided into three experimental groups as follows: Env formulated in AbISCO-100 (AbISCO) and ODN2395 (AbISCO+CpG) (n = 6), Env formulated in AbISCO (n = 6) and AbISCO and ODN2395 alone (Control) (n = 6). (A) Inoculations were given three times, at weeks 0, 8 and 32 (black arrows). Blood (red arrows), bone marrow (blue arrows), and vaginal and rectal lavage (green arrows) were sampled at the indicated time point. (B) Binding of Env-specific IgG represented as log10 of OD_50_ titers (left panel), and half-life during the long-term interval (right panel); each dot represent an animal and the lines represent a group, AbISCO+CpG (blue) and AbISCO (red). There was no difference in the Env-specific plasma antibody titers at any time point, as assessed by two-way ANOVA followed by Bonferroni multiple comparison post-test. (C, D) Mucosal responses presented as % Env-specific IgG of total IgG in the sample, were evaluated for vaginal (C) and rectal (D) lavages at four different time points (left panels) with error bars representing the standard error of the mean; AbISCO+CpG (blue) and AbISCO (red). Positive correlations between the mucosa Ab frequencies and the circulating Ab titers was found through non-parametric Spearman (r); linear regression indicates a good fit (right panels).

Previous studies have shown that the addition of TLR9 agonists can improve immune responses induced by commercially available human vaccines^4^,^5^. Here we investigated if a similar effect was observed when the TLR9 agonist CpG-C (ODN2395) was co-administered with the AbISCO, a TLR-independent saponin-based adjuvant, and purified HIV-1 Env glycoprotein. We first confirmed the stimulatory capacity of the CpG-C selected for the current study by *in vitro* stimulation of human and rhesus PBMCs, and compared it with CpG-C from other vendors. The results showed that the CpG-C batch used in the current study (purchased from Invivogen) stimulated equal or improved responses compared to CpG-C batches purchased from Sigma or Coley as determined by IgG secretion of stimulated cells (Supplementary Figure 1, left panel). We also confirmed that the CpG batch purchased from Invivogen was biologically active on rhesus cells in comparison to CpG-C purchased from Sigma or Hycult by testing its capacity to stimulate rhesus macaque memory B cells to differentiate into plasma cells as detected by B cell ELISpot analysis with positive results (Supplementary Figure 1, right panel). Having confirmed the functionality of the CpG-C batch we had selected for the *in vivo* experiments, we inoculated rhesus macaques divided into three groups as follows: gp140-F Env formulated in AbISCO and CpG-C (AbISCO+CpG) (n = 6), gp140-F Env formulated in AbISCO (n = 6) and AbISCO and CpG-C alone (Control) (n = 6). We did not include a group of animals that were inoculated with Env alone (no adjuvant) as we and others demonstrated previously that Env-specific antibody responses in the absence of adjuvant are low^24^,^25^. Furthermore, the inclusion of such a group was not critical for the objective of the current study, which was to investigate the role of TLR9 co-stimulation on the background of the Env-AbISCO formulation. The animals were inoculated three times, with an interval of two months between the first and the second immunization and an interval of 6 months between the second and the third immunization. The Env-specific IgG responses in plasma were evaluated two weeks after each immunization, as well as in the middle and at the end of the long interval and just prior to challenge (Figure 1A).

The kinetics of the Env-specific response was similar to that previously reported by our group^21^. All Env-immunized animals displayed a gp140-F-specific response two weeks after the first immunization and the OD_50_ titers reached peak levels two weeks after the second immunization. The responses then waned quite rapidly in the absence of boosting during the long interval, consistent with the half-life of IgG molecules and their origin from short-lived plasma cells; and after the third immunization reached peak titers again (Figure 1B left panel). There was no difference in the mean titers between the AbISCO+CpG and AbISCO groups at any of the time points (p > 0.05) (Figure 1B left panel, supplementary Figure 2) or in the half-life of the Env-specific IgG during the long interval, which was 2.6 weeks for the AbISCO+CpG group and 2.7 weeks for the AbISCO group (Figure 1B, right panel). These findings, together with our previously published results^21^, suggest that the cells responsible for most of the IgG production immediately after boosting are short-lived plasma cells generated from circulating memory B cells rather than continuously IgG secreting, long-lived plasma cells.

We next examined total and Env-specific IgG in vaginal and rectal lavages collected two weeks after each inoculation and just prior to the third inoculation. We found that the antigen-specific IgG in the lavages, plotted as Env-specific IgG of total IgG, were not significantly different between the AbISCO and the AbISCO+CpG groups, at any of the time points studied (p > 0.05). The rectal and vaginal Env-specific IgG responses displayed similar kinetics as that measured in plasma suggesting rapid redistribution of antibodies to the mucosa after boosting (Figure 1C and D). There was a high correlation between the Env-specific IgG levels in plasma and the mucosal compartments (vaginal r = 0.736, p < 0.0001; rectal r = 0.501, p = 0.0078) (Figure 1C and D, right panels) consistent with previous findings^21^. Collectively, the results showed that the kinetics and magnitude of the Env-specific IgG response in the periphery was correlated with the rectal and vaginal response, however none of those responses were enhanced by the addition of TLR9 co-stimulation to the formulation.

### Env-specific memory B cells and plasma cell responses are not enhanced by the addition of CpG-C to AbISCO

**Figure 2:**
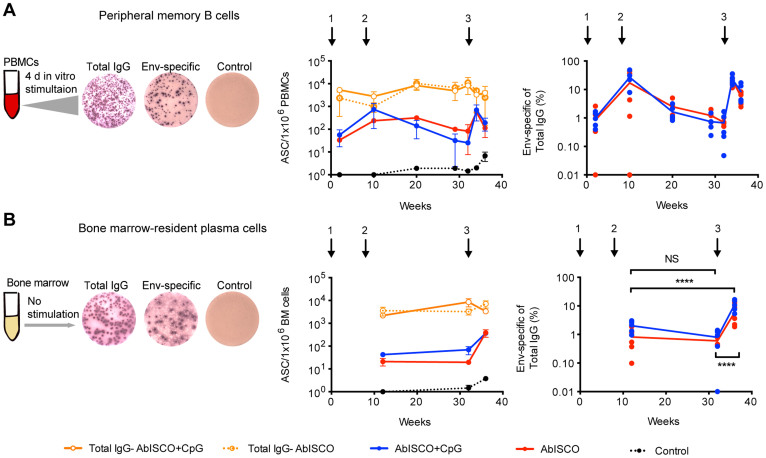
Env-specific ASC frequency in the periphery and in the bone marrow. A and B left diagrams show the experimental setup for the analysis of peripheral memory B cells (A) and bone marrow-resident plasma cells (B). Memory B cell analysis requires *in vitro* mitogenic activation for differentiation into ASC, contrary to plasma cells that spontaneously secrete Ab and already defined as ASC. Kinetics of the response plotted as ASC per 1 × 10^6^ cells (middle panels) and frequency of cells producing Env-specific IgG in relation to total IgG (right panels). Circles indicate group mean (n = 6). Quantification of the cells against: total IgG in the AbISCO group (orange circle, dotted line) and AbISCO+CpG group (orange circle, solid line); Env-specific in the AbISCO group (red) and AbISCO+CpG group (blue); and the negative control, Ovalbumin (black). The same color scheme was used in A and B. Because there was no difference between the AbISCO and AbISCO+CpG groups, these were pooled and responses at the different time points evaluated with Two-way ANOVA followed by Bonferroni's post-test for multiple comparisons. NS = non-significant. ****p < 0.0001.

T cell-dependent antibody responses give rise to high-affinity antigen-specific memory B cells and plasma cells that have their origin in the germinal center reaction. To more comprehensively evaluate the response against Env induced in the presence or absence of TLR9 co-stimulation, we next investigated the frequencies of antigen-specific cells in these two B cell compartments. We previously described a method to quantify total and antigen-specific rhesus macaques memory B cell frequencies by B cell ELISpot analysis after *in vitro* stimulation of peripheral blood mononuclear cells (PBMCs) followed by a short period of culturing to allow proliferation and differentiation of memory B cells into antibody-secreting cells^17^. Using this method, we enumerated total and Env-specific memory B cells in samples collected during the immunization schedule. In brief, one million PBMCs were stimulated with CpG-B, PWM and SAC and cultured for four days (Figure 2A, left panel) before B cell ELISpot analysis for either total Ab-secreting cells (ASC) or Env-specific ASC. The frequency of total IgG secreting cells was similar during all the time points tested with a mean of 5.4 × 10^3^ spots per million stimulated cells for both the AbISCO and AbISCO+CpG groups. The Env-specific memory B cell response followed that of the IgG response measured in plasma, reaching a plateau after the second immunization (AbISCO mean 236 and AbISCO+CpG mean 735 spots/million stimulated PBMCs; 17% and 27% Env-specific of total IgG, respectively), which declined rapidly and reached peak levels again after the third immunization (Figure 2A, middle panel). The addition of CpG-C did not further enhance the response induced by Env administered in AbISCO (Figure 2A, right panel).

In addition to a robust recall response, an effective vaccine should also elicit a durable Ab response that ideally should last years to decades after boosting to provide first-line defense against potential pathogen re-exposures. Such long-lived responses depend largely on Ab-secretion from bone marrow-resident plasma cells, which persist and secrete Ab for considerably longer periods of time than circulating, short-lived plasma cells in blood. The frequency of Env-specific plasma cells in bone marrow can be quantified after plating bone marrow cells directly into ELISpot plates without culturing the cells^17^,^21^. In previous studies, we reported Env-specific plasma cell frequencies in the bone marrow of around 3% of total IgG+ plasma cells after the second immunization with similar levels measured after the fourth immunization^21^. In the present study, the numbers of total IgG-producing plasma cells in the bone marrow were fairly constant over the study period with a mean of 4.7 × 10^3^ spots per million plated bone marrow cells. In contrast, the number of Env-specific plasma cells were similar at the first and second time points measured (after the first and second immunizations), but a clear increase in the numbers was measured after the third immunization (Figure 2B, middle and right panel). Similarly to the Env-specific plasma IgG responses and memory B cell responses, the addition of CpG-C did not enhance the frequencies of bone marrow-resident plasma cells induced by Env protein in AbISCO. Therefore, we pooled the mean frequencies of Env-specific plasma cells of total IgG-producing plasma cells for the AbISCO and AbISCO+CpG groups, and found a frequency of 1.4% and 0.7% at weeks 12 and 32, respectively, and 8.5% at week 36. The increase in frequency of Env-specific plasma cells at week 36 was statistically significant compared to the levels measured at week 12 and week 32 (p < 0.0001) (Figure 2B, right panel). These results illustrate that Env-specific ASC frequencies in the periphery and in the bone marrow followed a different pattern, which was not influenced by the addition of the TLR9 agonist. In sum, the addition of CpG-C to AbISCO did not detectably affect the magnitude of Env-specific circulating IgG, memory B cell or plasma cell responses in rhesus macaques.

### Env-specific CD4+ Th1 cell frequencies and cytokine secretion were not measurably affected by additional TLR9 stimulation

**Figure 3:**
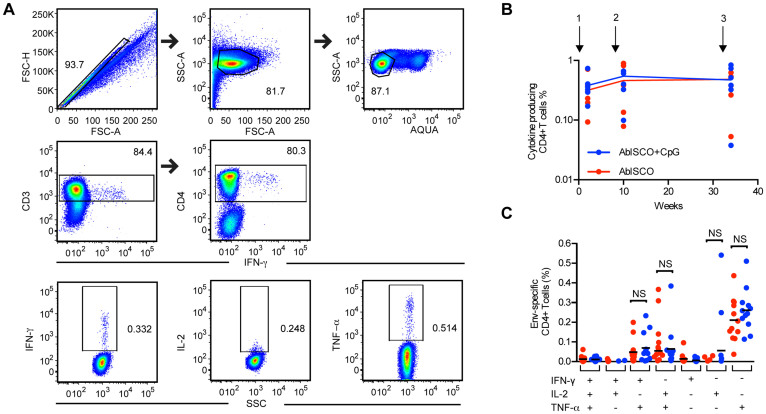
Env-specific CD4+ T cells frequency and cytokine secretion. Frequencies of cytokine-producing CD4+ T cells (IFN-γ, IL-2, TNF-α) after *in vitro* stimulation of PBMCs with 15-mer peptides spanning YU2 gp140-F. (A) Gating strategy from one representative animal (K17: in the AbISCO+CpG group) sampled two weeks after the second immunization is shown. Living, single lymphocytes (upper panels), were gated on CD3 and CD4 (middle panels) and then independently for IFN-γ, IL-2 or TNF-α positive populations (lower panels). (B) Kinetics of the frequency of cytokine producing Env-specific CD4+ T cells for each animal two weeks after the first, second and third immunization. Data from six individual animals were plotted after medium subtraction. (C) Total cytokine-producing Env-specific CD4+ T cells were subdivided in seven populations based on the production of one, two or three cytokines (IFN-γ, IL-2, TNF-α). Data from two weeks after the first and the second immunizations (n = 12) after medium subtraction are shown and analyzed with Mann-Whitney test. NS = non-significant.

One reason for the design of the current experiment was to determine if the addition of CpG-C would influence the frequency of Env-specific CD4+ T cells and/or their cytokine production profiles. We previously showed that both plasmacytoid dendritic cells (PDCs) and B cells from rhesus macaques are highly responsive to TLR9 stimulation including this particular CpG ODN similar to the corresponding human cell subsets^16^. We therefore evaluated the frequency and functional capacity of CD4+ T cells induced by Env administered in AbISCO, with or without CpG-C, for their capacity to secrete IL-2, TNF-α and IFN-γ in response to Env peptide stimulation. In brief, PBMCs from blood samples collected two weeks after the first, second and third immunizations were stimulated *in vitro* with a pool of 15-mer synthetic peptides spanning the full-length YU2 gp140-F trimer aa sequence. Cytokine production was assessed by multicolor flow cytometry and the gating strategy from one representative animal immunized with gp140-F is shown in Figure 3A. We found that cytokine-producing Env-specific CD4+ T cells were readily detected at each time point measured but there was no significant difference between the AbISCO+CpG and AbISCO groups (p > 0.05) (Figure 3B). The functional capacity of the Env-specific CD4+ T cells to produce one, two or three cytokines was also determined in terms of percentages, revealing that most T cells produced a single cytokine of the three measured, usually TNF-α (mean 0.24%). The frequency of cells simultaneously secreting two of the cytokines was 0.06% and 0.08% for IFN-γ+TNF-α+ and IL-2+TNF-α respectively (Figure 3C), and the frequency of cells secreting three cytokines was very low (0.01%). Based on these results, we conclude that Env-specific CD4+ T cells were similarly induced by both regimens and the quality of the Env-specific CD4+ T cell response was not measurably affected by the addition of CpG-C (p > 0.05).

### Neutralization activity against tier 1 and tier 2 pseudo-viruses was not influenced by addition of a TLR-9 agonist to AbISCO

**Figure 4:**
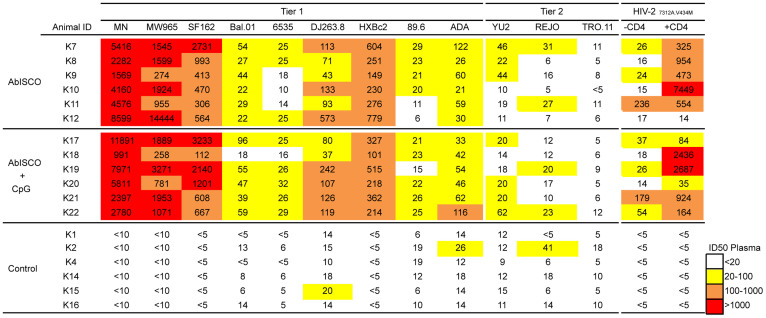
Plasma neutralizing activity against a panel of tier 1 and tier 2 Env-pseudoviruses. HIV-1 neutralizing activity in plasma collected two weeks after the third immunization was measured against a panel of tier 1 and tier 2 Env- pseudovirus isolates. Neutralization capacity against CD4-induced epitopes was measured in the presence and absence of soluble CD4. Data from individual animals are shown as the reciprocal dilution giving 50% neutralization (ID_50_ titer). Color scale is given according to the ID_50_ neutralization titer of plasma with white representing the absence of neutralization, yellow moderate, orange medium and red high.

**Figure 5:**
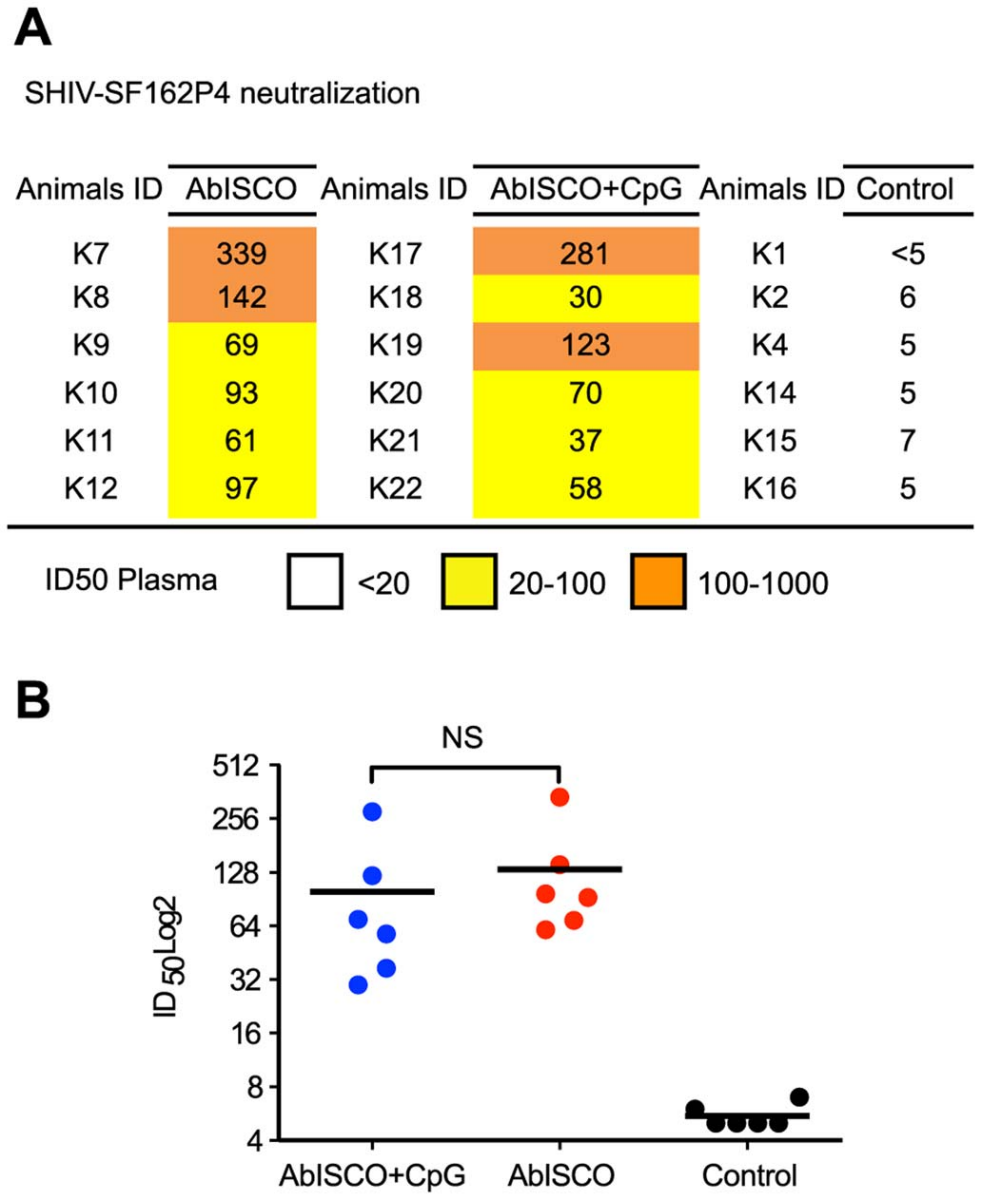
Plasma neutralization of the SHIV-SF162P4 challenge stock. Neutralizing activity against the SHIV-SF162P4 challenge stock was determined in plasma sampled two weeks following the third immunization. (A) Data from individual animals are shown as the reciprocal dilution giving 50% neutralization (ID_50_ titer). (B) Sera from animals in both the AbISCO and AbISCO+CpG groups displayed significantly higher neutralizing Ab titer against SHIV-SF162P4 compared to Control (*p < 0.05, **<0.01) with no difference between the groups (p > 0.05). Statistics were evaluated with the Kruskal-Wallis test followed by Dunn's test for multiple comparisons. NS = non-significant.

Due to the extreme genetic variation of HIV-1, the induction of antibodies capable of neutralizing diverse sets of HIV-1 variants is a high priority. The addition of CpG to alum-based adjuvants was shown to increase the functionality of the specific Ab response in terms of affinity and neutralization capacity^5^,^26^. In the current study, having demonstrated that the Env binding titers were not modulated by the addition of CpG, we evaluated the capacity of plasma collected two weeks after the third immunization to neutralize a panel of HIV-1 Env-pseudotyped virions. Neutralization breadth was assessed by including neutralization-sensitive viruses (tier 1) as well as neutralization resistance primary circulating variants (tier 2) in the panel. The data are represented as 50% plasma inhibitory dilution (ID_50_) values. In agreement with previous Env immunogenicity studies performed by us or others, we observed potent ID_50_ neutralizing titers against tier 1 viruses and only modest activity against tier 2 viruses^17^. However, compared to our previous studies where we used shorter injection intervals^17^, in the present study we detected ID_50_ neutralizing titers against several tier 2 viruses, suggesting an improvement in the response. For example, neutralization of viruses pseudotyped with the neutralization resistant homologous YU2 was observed in 7 of 12 animals. A similar pattern was observed for REJO (Figure 4). High ID_50_ neutralizing titers were detected against the HIV-2_7312A.V433M_ virus in the presence of soluble CD4 (sCD4), an assay that is diagnostic of co-receptor binding site (CoRbs)-directed Abs^27^. This is consistent with our previous results where we showed that CoRbs-directed Abs are readily induced in non-human primates by Env immunogens capable of binding primate CD4^28^. There was no statistically significant difference in ID_50_ neutralizing titers between Env-inoculated animals in the AbISCO+CpG and AbISCO groups, suggesting that the capacity to induce neutralizing Abs against HIV-1 was not influenced by the addition of CpG-C (p > 0.05) (Supplementary Figure 3). We also asked if the pre-challenge plasma contained Abs capable of neutralizing the SHIV-SF162P4 challenge stock. Moderate ID_50_ neutralization titers ranging between 100 and 300 were measured against the stock (Figure 5A), similar to what was observed in previous experiments by us and others^17^,^29^,^30^. Again, these levels were similar between Env-inoculated animals in the AbISCO+CpG and AbISCO groups (Figure 5B).

### Improved control of viremia following SHIV challenge in Env-immunized animals

We next assessed the impact of TLR9 co-stimulation for the induction of protective responses in the context of SHIV challenge. It remained possible that immune parameter(s) that we had not examined in the assays described above differed between the AbISCO+CpG and AbISCO groups, such as antibody-mediated cytotoxicity (ADCC), shown to be associated with protection against SIV infection^31^. To investigate a potential protective effect of the vaccine-induced responses, we challenged Env-immunized animals with SHIV-SF162P4 via the vaginal route, four weeks after the last immunization, with a single dose of 1 ml, corresponding to 100 TCID_50_ (tissue culture infectious dose-50). In addition to the two groups of Env-immunized animals, Env-naïve animals from two control groups were similarly challenged; one group consisting of the six animals injected with AbISCO+CpG (no Env), and one group consisting of 5 animals that had not received any prior injections. Viral replication in plasma was monitored at frequent intervals for seven weeks after the challenge, after which time the SHIV-SF162P4 infection was naturally suppressed in all control animals. There was no difference in the kinetics or accumulated viral loads between the two groups of control animals (Supplementary Figure 4) and these were therefore pooled for the subsequent analyses of the challenge experiment.

**Figure 6:**
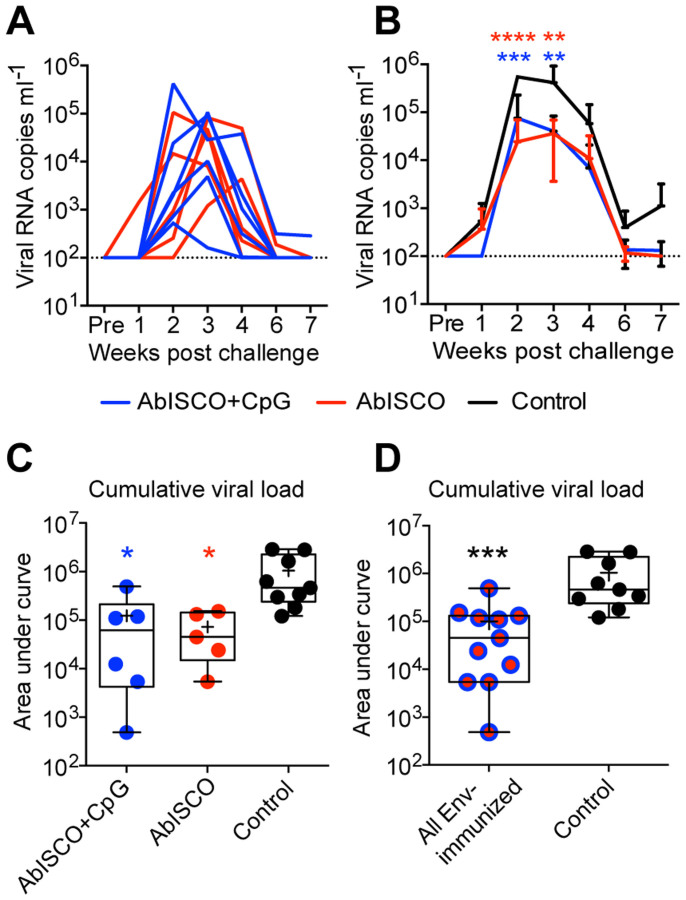
Evaluation of SHIV-SF61P4 viral load in infected macaques. Viral load (copies per ml) was determined by one-step Q-RT-PCR in plasma sampled weekly, for seven weeks, following vaginal challenge with 100 TCID_50_ SHIV-SF162P4 viruses. The limit of detection was 100 viral RNA copies per ml plasma. (A) Viral load curves are shown for individual Env-immunized macaques in the AbISCO (red, n = 5) or AbISCO+CpG (blue, n = 6) groups. (B) Group viral load curves are shown (mean ± SD) for AbISCO (red n = 5), AbISCO+CpG (blue, n = 6) and Control (black, n = 9) over a seven week period. Statistical differences were evaluated with Two-way ANOVA followed by Bonferroni's post-test. Red stars indicate AbISCO vs Control (2 w post challenge, p < 0.00001; 3 w post challenge, p = 0.0053) and Blue stars indicate AbISCO+CpG vs Control (2 w post challenge, p = 0.0.0001; 3 w post challenge, p = 0.0032). (C, D) The cumulative viral load over the seven weeks of monitoring was determined by quantifying the area under curve. In (C) the AbISCO (red) and AbISCO+CpG (blue) groups were compared with the Control group and in (D) the pooled AbISCO and AbISCO+CpG groups, referred to as “All Env-immunized” (red/blue circles, n = 11) were compared with the Control group (black, n = 9) using the Kruskal-Wallis test followed by Dunn's post-test (C; p = 0.0321 and p = 0.0155 respectively) and the Mann-Whitney test (D; p = 0.0005). Significant differences were determined as *p < 0.05, **p < 0.01, ***p < 0.001 and ****p < 0.0001. Box plots show median (line), mean (plus), 25–75% (box) and min-max (whiskers). The same color scheme was used throughout the figure.

When the Env-immunized animals were examined, we observed similar viral load (copies/ml) curves in the plasma of all animals whether in the AbISCO+CpG or AbISCO groups (Figure 6A). Two Env-naïve animals in the control group and one Env-inoculated animal in the AbISCO+CpG group did not become infected for reasons we did not investigate further in the current study. We excluded the non-infected animals from further analysis to focus on the effect of Env-specific Abs on the viral titers and cumulative viral loads in the animals that were infected. When the SHIV viremia in the Env-vaccinated animals was analyzed relative to the control animals, it was apparent that vaccination resulted in improved control with statistically lower viral loads between the groups at several time points (Figure 6B). There was also a significant control of viremia as measured by the viral copy number as seen by the reduced cumulative vial load when comparing the Env-immunized groups with the Env-naïve control group (Figure 6C). This difference was even more pronounced when all Env-immunized animals were combined in one group and compared to the Env-naïve control animals (Figure 6D) using a non-parametric Mann-Whitney test for statistical calculations (***p = 0.0005). The control of viremia observed in the Env-immunized animals was not influenced by the inclusion of CpG-C in the formulation (p > 0.999). Overall, these results demonstrate the potential of administering Env in AbISCO to enhance the control of viremia after heterologous vaginal SHIV-SF162P4 challenge.

## Discussion

Synthetic TLR ligands are frequently used as vaccine adjuvants in pre-clinical and clinical studies^10^,^11^,^12^,^13^. In our current immunization study in rhesus macaques, we investigated if immune responses elicited by soluble HIV-1 Env formulated in AbISCO were enhanced by the addition of a TLR9 ligand. We examined B and T cell responses in animals inoculated three consecutive times with Env in AbISCO, with or without the addition of CpG-C, using a relatively long interval of six months between the second and third inoculation. We show that high Env-specific Ab titers were induced in both groups, with no detectable difference in either the induction or contraction of response kinetics. Similarly, there was no difference between the two groups of animals in terms of the frequencies of Env-specific peripheral memory B cells, bone marrow-resident plasma cells, HIV-1 neutralizing Ab responses or control of viremia after SHIV-SF162P4 challenge. This does not exclude the possibility that the addition of CpG-C modulated the Env-directed immune response in ways that were not detectable in our assays. For example, it may have altered the fine sub-specificities of elicited response or the Ab V(D)J gene segment usage, as reported in a recent study using *Plasmodium vivax* protein antigen administered in an oil-in-water formulation in the presence or absence of various TLR ligands^12^.

Somewhat surprisingly, we also observed similar frequencies and cytokine production profiles of Env-specific CD4+ T cells in both groups. This contrasts to the increased frequency of IFN-γ-producing CD4+ T cells observed in mice inoculated with soluble cytomegalovirus gB protein formulated in AbISCO with CpG compared to gB protein administered in AbISCO alone^32^. This difference might be explained by the fact that mice are more responsive to CpG compared to primates due to their broader tissue expression of TLR9^33^. Another possibility is that CpG is not active in the context of HIV-1 Env in primates due to interference with TLR9 signaling in PDCs as suggested in a recent report using human PDC cultures^34^. However, a recent study by Moody and co-workers found that the addition of a TLR9 agonist to an Env vaccine significantly enhanced tier 1 neutralizing antibodies and ADCC compared to other TLR agonist combinations suggesting that CpG has an effect *in vivo* when co-administered with Env^35^. Instead, we consider it more likely that the lack of an effect observed by the addition of CpG-C in our experiments is because the formulation of Env in AbISCO induces a well-balanced immune response on its own. Of note, a recent human trial using influenza virosomes adjuvanted in clinical grade AbISCO (Matrix-M) reported that a balanced Th1/Th2 response was induced^36^. Thus, it is possible that, unlike for Th2-promoting alum-based adjuvants where positive effects of adding TLR9 ligands were observed^4^,^23^, TLR9 co-stimulation is not required to enhance or broaden the adjuvant effect of AbISCO.

The choice of adjuvant for an optimal protein-based HIV-1 vaccine remains an important issue. ISCOMATRIX and AbISCO belong to the new generation of immune-stimulating complexes (ISCOM) adjuvants, which are based on the Quillaja saponin mixed with cholesterol and phospholipids at defined ratios. These adjuvants possess both immunostimulatory and delivery properties and antigen is co-administered rather than incorporated into the ISCOMs themselves^37^. Matrix adjuvants are used in several registered animal vaccines and a human clinical trial with Matrix-M, which is analogous to AbISCO, was recently completed^36^. ISCOMATRIX-based adjuvants were shown to induce a balance Th1/Th2 response, NK cell activation, cross-presentation to CD8+ T cells and long-lasting Ab and T cell responses^38^,^39^. The mechanism of immune-stimulation is not well understood; however, studies have shown that tissue-resident antigen presenting cells at the site of injection internalize the adjuvant, resulting in endosomal stress followed by apoptosis, which in turn triggers infiltration of monocytes and neutrophils that transport the antigen to draining lymph nodes for T and B cell activation^39^,^40^. In the primate immune system, TLR9 is expressed primarily by PDCs and memory B cells and these cells respond readily to CpG^41^,^42^,^43^. *In vivo* and *in vitro* studies have established that PBMCs from rhesus macaques respond similarly, demonstrating that rhesus macaques are suitable for evaluation of TLR9 ligands, including the specific ligand (CpG-C) used here. However, differences between human and macaque responses to TLR9 stimulation also exist, for example CpG-C is more potent than CpG-A or CpG-B to induce B cell proliferation in rhesus PBMC cultures, while CpG-B stimulates more potent B cell proliferation in human cultures^16^. In our current study, as well as in our previous Env immunogenicity studies in non-human primates, we therefore used CpG-C^17^.

The need to improve existing Env-based vaccines has spurred much activity in the field, both to design improved Env trimers and to identify effective adjuvants. The ultimate goal of Env-based immunization is to induce Abs with the potential to protect against highly diverse circulating HIV-1 strains. While this goal so far remains elusive, we propose that comprehensive evaluation of candidate adjuvant formulations in non-human primates can be performed side-by-side with Env immunogen design and evaluation, as shown here. The main challenge to elicit bNAbs is likely the design of well-ordered trimers that retain the native conformation of the functional, trimeric Env spike, such as the recently presented BG505 trimers^44^,^45^. The evaluation of such trimers in vivo will provide important information about how antigenicity translates into immunogenicity and the elicitation of Abs targeting bNAb epitopes. In parallel, existing Env immunogens, such as the rather open conformation of the gp140-F trimers used here^46^, can be used as a model Env immunogens to address more general questions about the response, such as the development of antigen-specific memory B cells and plasma cells in response to different adjuvants. A direct comparison to evaluate different adjuvants induced by HIV-1 Env trimer inoculation in guinea pigs show that Matrix-M was one of the most effective adjuvants tested^47^. Similar side-by-side studies aimed at evaluating the use of different adjuvants with soluble Env in non-human primates are critically needed for best prioritization of future Env immunogenicity studies.

Durable responses is a goal of all prophylactic vaccines^48^. Long-lived plasma cells residing in the bone marrow are responsible for the maintenance of long-term antibody responses after infection or vaccination. However, studies of these cells are hampered by the challenge of access to the bone marrow compartment. Here, we demonstrate a significantly higher frequency of Env-specific plasma cells after the third immunization following the long-term interval, compared to after the second immunization. The source and longevity of these plasma cells needs to be addressed in subsequent studies. The processes that regulate the generation of long-lived plasma cells remain insufficiently understood. Recent data from two different studies show that alum and TLR-based adjuvants differentially imprint plasma cell survival through programs taking place in late germinal center B cells^49^,^50^. The implications in the context of our results highlight the need to identify adjuvants and/or immunization regimens that affect plasma cell differentiation and survival programs to improve the durability and quality of vaccine-induced responses.

In summary, our results demonstrate that administration of HIV-1 Env formulated in AbISCO, using an immunization interval similar to that used for the human Hepatitis B virus vaccine, induced Env-specific B and T cell responses and an improved control of SHIV viremia in rhesus macaques compared to unimmunized control animals or animals inoculated with adjuvant alone. TLR9 co-stimulation did not further enhance the Env-specific immune responses or the control of viremia, suggesting that AbISCO/Matrix-M has the potential to act as an effective stand-alone adjuvant for protein-based vaccines.

## Methods

### Ethics statement

The animal work was conducted with the approval of the regional Committee for Animal Ethics (Stockholms Norra djurförsöksetiska nämnd). All methods were carried out in accordance with the approved guidelines.

#### Animals

Rhesus macaques (Macaca Mulatta) of Chinese origin, approximately 5–6 years old, were housed at the Astrid Fagraeus Laboratory facility at Karolinska Institutet. Housing and care procedures were in compliance with the provisions and general guidelines of the Swedish Board of Agriculture, and the facility has been assigned an Animal Welfare Assurance number by the Office of Laboratory Animal Welfare (OLAW) at NIH. The macaques were housed in pairs in 4 m^3^ cages, enriched to give them possibility to express their physiological and behavioral needs. They were habituated to the housing conditions for more than 6 weeks before the start of the experiment, and subjected to positive reinforcement training in order to reduce the stress associated with experimental procedures. All immunizations and blood samplings were performed under sedation with ketamine 10 mg/kg intramuscularly (i.m.) (Ketaminol 100 mg/ml, Intervet, Sweden) and when the macaques were sampled for rectal or vaginal lavages, or bone marrow, to induce total muscle relaxation and analgesia, they were given an additional 0.5 mg/kg i.m. Xylazine (Rompun®, Bayer, Sweden). The macaques were weighed at each sampling. Before entering the study, all animals were confirmed negative for simian immunodeficiency virus (SIV), simian T-cell lymphotropic virus and simian retrovirus type D.

#### Expression and purification of Env immunogens

Soluble gp140-F trimers were produced by transient transfection of Freestyle 293F suspension cells (Life Technologies) as previously described^28^. In brief, cells were transfected at a density of 1.1 × 10^6^/ml in GIBCO®Freestyle293 expression media using 293Fectin, according to manufacturer's instructions (Life Technologies). Supernatants were collected four days after transfection. Following collection, all supernatants were centrifuged at 3,500 g to remove cells or cell debris, filtered through a 0.22 μm filter and supplemented with Complete™, EDTA-free protease inhibitor cocktail (Roche) and Penicillin-Streptomycin (Life Technologies) and stored at 4°C until further purification. First, the proteins were captured via glycans by lentil-lectin affinity chromatography (GE Healthcare, Uppsala, Sweden). After extensive washing with PBS, supplemented with 0.5 M NaCl, the proteins were eluted with 1 M methyl-α-D-mannopyranoside and captured in the second step via the His-tag by nickel-chelation chromatography (GE Healthcare). Following a wash with 40 mM imidazole (IM) and 0.5 M NaCl in PBS, proteins were eluted with 300 mM IM in PBS. Trimers were then separated from lower molecular weight forms by gel filtration chromatography using a superdex200 26/60 prep grade column by the ÄKTA Fast protein liquid chromatography system (GE Healthcare). Biotinylated Env probes used in the B cell ELISPOT assay were purified by lentil-lectin and nickel-chelation chromatography but not subjected to subsequent gel filtration chromatography as previously described^28^. Env probes were biotinylated via site-directed biotinylation of an Avi-tag sequence using biotin ligase enzyme according to the manufacturer's instructions (Avidity). Correct folding of the Env protein was verified by successful binding to monoclonal antibodies, VRC01, 17b, 39F, 447, GE148 and 2G12 by ELISA.

#### Immunizations and sampling

Two experimental groups of animals were inoculated three times with soluble gp140-F Env trimers in combination with 75 μg AbISCO (Isconova AB, now Novavax) for one group, or 75 μg AbISCO with 500 μg CpG ODN2395 (Invivogen) to the other group. Env protein was given at 200 μg per animal for the first inoculation and 100 μg for the following injections. The control group was inoculated with 75 μg AbISCO and 500 μg CpG ODN2395 only. We selected this dose of CpG based upon previous studies in rhesus macaques^51^ and humans^4^. Inoculations were performed at weeks 0, 8 and 32 by the i.m. route of injection, given in a total volume of 1 ml, divided equally between the left and right hind leg. Blood samples were taken at eight different times, vaginal and rectal samples were taken at four different times and bone marrow samples were taken at three different time points (Figure 1A). Blood, bone marrow and lavages were sampled as previously described^17^,^52^. PBMCs were separated from EDTA blood by Ficoll-Paque™ PLUS (GE Healthcare). Bone marrow cells were treated with red-blood cell lysis and frozen in FCS in 10% DMSO at −150°C.

#### Analysis of Env-binding Abs

HIV-1 Env-specific IgG titers from plasma, vaginal or rectal lavages were measured by ELISA as previously described^53^ with the following modifications. Briefly, gp140-F trimer, strain YU2, was coated onto Nunc Maxisorp microtiter plates at 100 ng/well in PBS ON at +4°C. After blocking in PBS containing 2% non-fat dry milk, samples were added and incubated for 1.5 h at 37°C. The gp140-F specific IgG was detected by adding secondary HRP conjugated anti-monkey IgG (Nordic Immunology, Tillburg, The Netherlands) and the signal developed by addition of TMB+ (Invitrogen). Reactions were terminated by adding an equal volume of 1 M H_2_SO_4_ and the optical density (OD) was read at 450 nm. Between each incubation step, the plates were washed 6 times with PBS supplemented with 0.05% Tween 20. The half-max binding titers (OD50) for each sample was calculated by interpolation from mean OD50 values calculated from an immunized plasma using the formula (ODmax-ODmin)/2).

#### Total IgG in supernatant from human stimulated PMBCs

Human PBMCs were stimulated for 7 days with 5 μg/mL CpG-C (ODN2395) from Invivogen, Sigma, or Coley pharmaceuticals in 96-well cultures at 1 × 10^6^ cells/mL. IgG antibody levels in the supernatant were then measured as previously described^17^. Briefly, anti-IgG (2 μg/mL) (Jackson Immunoresearch) was coated onto Nunc Maxisorp microtiter plates in PBS ON at +4°C. After blocking in PBS containing 2% non-fat dry milk, supernatants and standard were added and incubated for 1 h at 37°C. Total IgG was detected by adding secondary HRP conjugated anti-human IgG and the signal developed by addition of TMB+ (Invitrogen). After 5 minutes the reaction was stopped by adding an equal volume of 1 M H_2_SO4. The optical density (OD) was read at 450 nm.

#### B cell ELISpot assay

To evaluate the frequency of antigen specific memory B cells, total PBMC were cultured at 2 × 10^6^ cells/ml and stimulated with CpG-B (ODN-10103; Coley) or CpG-C (ODN-2395; Invivogen) (5 μg/ml), pokeweed mitogen (PWM, 10 μg/ml, Sigma), and *Staphylococcus aureus* Cowan strain (SAC, 1:10000, Sigma) for 4 days in 48-well plates. As the detection of antigen specific plasma cells does not require stimulation, bone marrow cells were plated directly into ELISpot plates at 10 × 10^6^ cells/ml. To detect Ab-secreting cells, MAIPSWU10 96 well plates (Millipore) were coated with 10 μg/ml anti-human IgG (Fcγ) (Jackson ImmunoResearch) and previously stimulated PBMCs or unstimulated bone marrow cells were transferred to the plates in dilution series and incubated ON at 37°C, 5% CO2. The plates were then washed with PBS containing 0.05% Tween and incubated with biotinylated gp140-F (strain YU2) followed by washing and incubation with streptavidin-AP (1:1000, Mabtech). The reactions were developed using BCIP/NBT substrate (Sigma) and stopped by washing in water. Spots corresponding to Ab-secreting cells (ASC) were counted using an ImmunoSpot^R^ analyzer (Cellular Technology Ltd.). The results were converted to Ab secreting cells per million cultured PBMCs or plated bone marrow cells (ASC/10^6^ cells), and the frequency of antigen-specific cells was calculated from the total number of ASC.

#### Intracellular cytokine staining of Env-specific T cells

Frozen PBMCs were thawed and rested ON in medium at 37°C with 5% CO_2_. Cell viability was consistently >90%. Cells were added at 1 × 10^6^ cells/well in medium alone (RPMI 1640 medium containing 10% FCS, 2 mM L-glutamine, 100 U/ml penicillin, 100 mM streptomycin, 2% HEPES, all from Life Technologies), or in medium containing 1 μg/ml Staphylococcal enterotoxin B (SEB) or 2.5 μg/ml Env peptides. The Env peptide pool consisted of 15-mer peptides, overlapping by 10 amino acids (aa), spanning the full-length gp140-F sequence (New England Peptide LLC). All cultures contained anti-CD28 and anti-CD49d at 1 μg/ml with Brefeldin A (10 μg/ml) (Becton Dickinson). Cells were incubated for 6 h in 5% CO_2_ at 37°C, washed and surface stained with CD8-Alexafluor 700 clone RPA-T8 and CD4-APC clone L200. Cells were then permeabilized using the cytofix/cytoperm kit (Becton Dickinson) and stained with CD3-Pacificblue clone SP34-2, IL2-PE clone MQ1-17H, IFN-γ-FITC clone B27 and TNF-α-PECy7 clone MAB11. All Abs were from BD-Pharmingen and were previously titrated for optimal staining of rhesus macaque PBMCs. Cell viability was assessed following staining with the Live/Dead Fixable Aqua Dead Cell Stain Kit (Molecular Probes). Samples were collected on a FACS LSRII (BD Immunocytometry Systems) and analyzed using FlowJo software (Tree Star), Pestle v1.7 and SPICE v5.1 (Mario Roederer, Vaccine Research Center, NIH, USA).

#### Virus neutralization assays

Plasma samples were taken from immunized animals two weeks after the third immunization and tested for virus neutralization capacity against a panel of diverse HIV-1 isolates and the SHIV-SF162P4 challenge stock. Neutralization assays were performed using a single round of infection HIV-1 Env pseudovirus assay or replication competent virus with protease inhibitors, and TZM-bl target cells as previously described^54^,^55^,^56^. Env pseudoviruses were prepared by co-transfecting 293T cells with an Env expression plasmid containing a full gp160 *env* gene and an *env*-deficient HIV-1 backbone vector (pSG3ΔEnv). To determine the plasma dilution that resulted in a 50% reduction in RLU, serial dilutions of sera were performed and the neutralization dose-response curves were fit by non-linear regression using a 4 parameter hill slope equation programmed into JMP statistical software (JMP 5.1, SAS Institute Inc., Cary, NC). The results are reported as the plasma neutralization ID_50_, which is the reciprocal of the dilution producing 50% virus neutralization. Diverse HIV-1 virus isolates, including viruses from clades A, B and C were used in the neutralization assays. Clade B viruses included a panel of Env pseudoviruses that were recently characterized and recommended for use in assessing neutralization by HIV-1 immune sera^54^. Replication-competent viruses or functional Env plasmids for pseudoviruses were originally obtained from Dana Gabuzda (YU2 and MuLV), Leo Stamatatos (SF162) and James Binley (JRFL), respectively. The clade A DJ263.8 sequence was cloned from the original PBMC derived virus provided by Francine McCutchan and Vicky Polonis (U.S. Military HIV Research Program) and the clade C MW965 Env plasmid was obtained from the AIDS Research and Reagent Repository. Isolation of the Env plasmids BaL.01 were described^55^ as was the SS1196.1 Env^54^.

#### SHIV challenge

The gp140-F (n = 12) and adjuvant only (n = 6) inoculated macaques were challenged intravaginally with 100 TCID_50_ (tissue culture infectious dose-50) SHIV-SF162P4 diluted in 1 ml PBS four weeks after the third immunization. Viral loads were monitored weekly by Q-RT-PCR, with a cut-off of 100 copies per ml, for 7 weeks following challenge. The no-adjuvant Control (n = 5) had been challenged at an earlier time point. The SHIV-SF162P4 challenge stock was derived from an acutely infected rhesus macaque as described previously^29^,^57^. The virus induces transient viremia in infected animals and is in most cases cleared upon production of virus-specific neutralizing antibodies^29^,^58^. Six weeks prior to challenge, all animals received 30 mg Depo-provera to normalize the vaginal mucosa.

#### Q-RT-PCR

Viral RNA was isolated from 500 μl plasma using the MiniAmp vacuum kit (Qiagen) and eluted in 50 μl water. Gag-specific RNA was quantified immediately following RNA isolation by assaying 10 μl RNA in 20 μl reactions with the qScript one-step fast qRT-PCR low ROX kit (Quanta biosciences) using primers at 400 nM and probes at 80 nM as described previously^17^,^59^. An RNA standard, previously generated from SIVmac239 Gag^17^, was included in all experiments at a 10-fold dilution series, ranging from 10^6^ to 10^1^ RNA copies. All experiments were performed on an Abi Prism 7500 fast cycler (Life Technologies). The detection limit of the assay was 10 RNA copies per reaction, translating to 100 RNA copies per ml plasma. PCR efficiency was 99.9 ± 4.4% (average ± SD; n = 15) with an r^2^ of 0.996±0.004 (average ± SD; n = 15).

#### Statistical analysis

In all group comparisons including ≥3 groups, statistical significances were determined by the non-parametric Kruskal-Wallis test followed by Dunn's post-test for individual comparisons. When comparing <3 groups the Mann-Whitney test was used. For simultaneous evaluation of statistical differences between samplings and groups over the course of the study, two-way ANOVA was used followed by Bonferroni's post-test for multiple comparisons. Analysis of relationship between variables was done with the non-parametric Spearman's correlation analysis. All statistical analysis was done with Graph Pad Prism software version 5 or 6 and considered significant at * for p ≤ 0.05, ** for p ≤ 0.01, *** for p ≤ 0.001 and **** for p ≤ 0.0001.

## Author Contributions

C.S., R.T.W. and G.B.K.H. designed the study. R.T.W. and G.B.K.H. contributed reagents. P.M., C.S. and S.O. performed the research. C.S., P.M., J.R.M. and G.B.K.H. analyzed the data. C.S., P.M. and G.B.K.H. wrote the paper.

## Supplementary Material

Supplementary Information Supplementary Information
