# Supplementary material for: Primate immune responses to HIV-1 Env formulated in the saponin-based adjuvant AbISCO-100 in the presence or absence of TLR9 co-stimulation
Source: Sci Rep. 2015 Mar 12;5:8925. doi: 10.1038/srep08925 (PMC4356977; doi:10.1038/srep08925)
Supplement: Supplementary Information [file srep08925-s1.pdf]

Supplementary material

Primate immune responses to HIV-1 Env formulated in the saponin-based adjuvant AbISCO-100 in the presence or absence of TLR9 co-stimulation

Paola Martinez[1,#], Christopher Sundling[1,#,§], Sijy O'Dell[2], John R. Mascola[2], Richard T. Wyatt[3], and Gunilla B. Karlsson Hedestam[1,*]

[1]Department of Microbiology, Tumor and Cell Biology, Karolinska Institutet, Stockholm, Sweden, [2]Vaccine Research Center, National Institute of Allergy and Infectious Diseases, National Institutes of Health, Bethesda, MD, USA and [3]Department of Immunology and Microbial Science, IAVI Neutralizing Antibody Center, The Scripps Research Institute, La Jolla, CA USA

[§]Current address: Immunology division, Garvan Institute of Medical Research, Darlinghurst, NSW, Australia.

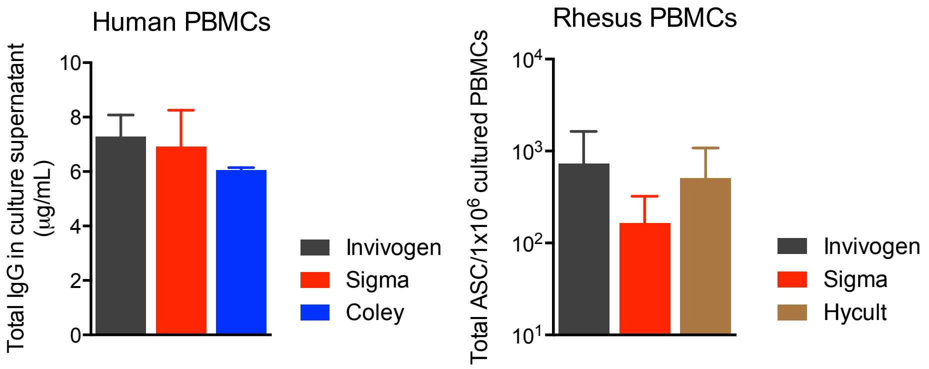

Supplementary Figure 1. Assessment of the stimulatory capacity of the TLR9 agonist CpG-C (ODN 2395) used in the current study (Invivogen) in comparison with CpG-C from other sources (Sigma, Coley Pharmaceuticals and Hycult). The CpG-C stimulatory capacity was assessed by culturing human and rhesus PBMCs with 5 □g/mL CpG-C followed by measurements of IgG antibody in the supernatants or IgG+ ASC by ELISA and ELISpot analysis respectively. Shown is the mean +SEM of 2 donors.

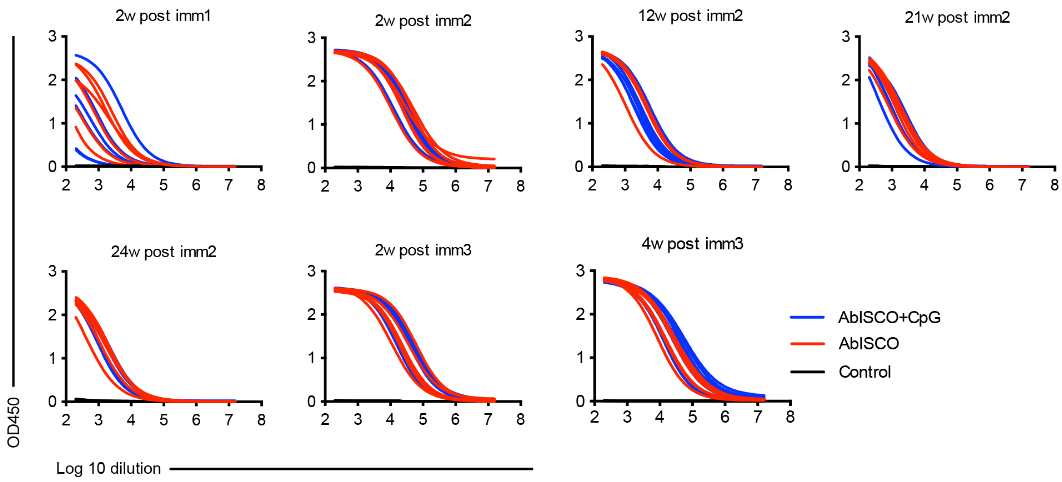

Supplementary Figure 2. Titration curves of Env-specific IgG responses for the individual animals represented as the optical density against the log 10 reciprocal plasma dilution. Every graph represents a different time point after inoculation. AbISCO (red) and AbISCO+CpG (blue).

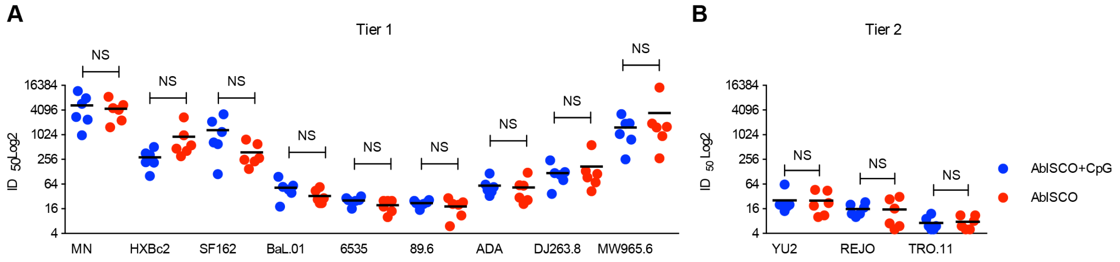

Supplementary Figure 3. Comparison of Ab neutralization activities against a panel of tier 1 (A) and tier 2 (B) Env-pseudoviruses elicited by Env administered in AbISCO alone (red) or AbISCO+CpG (blue). Plasma was collected two weeks after the third immunization. Data from individual animals are shown as the reciprocal dilution giving 50% neutralization (ID50 titer) and statistics was determined with the Mann-Whitney test. NS= non-significant.

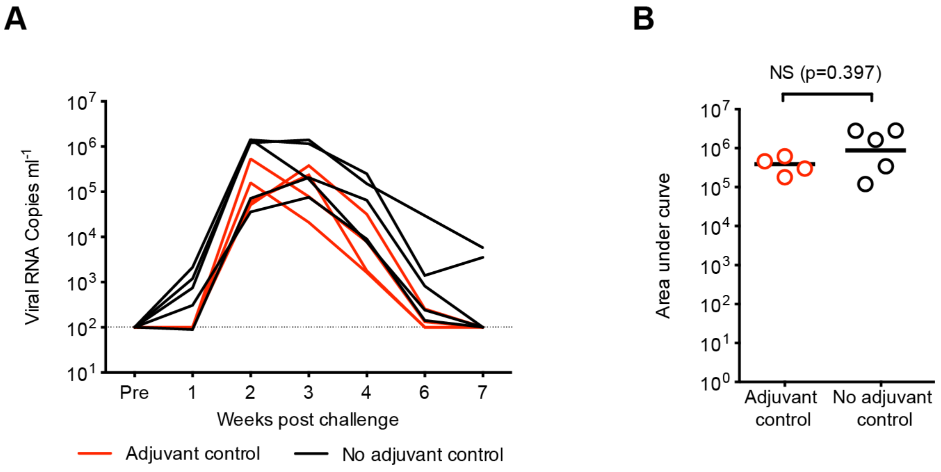

Supplementary Figure 4. Comparison of viremia after SHIV-SF162P4 challenge of animals in the two control groups: one group consisting of six animals injected with AbISCO+CpG (adjuvant control; red), and the other group consisting of 5 animals that had not received any previous injection (No adjuvant control; black). Kinetics of the viral replication (copies per mL) after the SHIV challenge (A) and accumulated viral loads (B) for both sets of control animals. Statistics was determined with the Mann-Whitney test. NS= non-significant.
